# Supplementary material for: Cellular eEF1G Inhibits Porcine Deltacoronavirus Replication by Binding Nsp12 and Disrupting Its Interaction with Viral Genomic RNA
Source: Viruses. 2025 Oct 13;17(10):1369. doi: 10.3390/v17101369 (PMC12568264; doi:10.3390/v17101369)
Supplement: Supplementary file 1 [file viruses-17-01369-s001.zip › Table S2.pdf]

Table S2. Primers used for PCR

Target Gene	Primers	Primer Sequences（5’-3’）
Strand-specific RT-qPCR	RT-Negative	TCATGGTGGCGAATAAGCATTAGCGGCTTGTGGTTT
	RT-β-actin	CTAGAAGCATTTGCGGTGGAC
PDCoV negative RNA	negative-F	TCATGGTGGCGAATAA
	negative-R	TCACCCGGTAGGGGGTTTTA
porcine β-actin	β-actin-F	ACCACCATGTACCCAGGCAT
	β-actin-R	GGACTCGTCGTACTCCTGCT
PDCoV positive RNA	positive-F	GCCATCAAGTTGGCTACCCT
	positive-R	ACCCTACGGTAGTCAGTGCT
PDCoV sgmRNA	sgmRNA-F	AATTTTATCTCCCTAGCTTCG
	sgmRNA-R	GTAGCCAGGAAGAACGCCAACA
PDCoV 5’UTR	5’UTR-F	CTCCCTAGCTTCGCTAGTTCTCT
	5’UTR-R	CAGTGCTGTCTATAGAAGTGGTGGA
pCAGGS-HA-5’UTR	HA-5’UTR-F	gttccagattacgctgaattcACATGGGGACTAAAGATAAAAATTATAGC
	HA-5’UTR-R	attaagatctgctagctcgagTTTCACAGCTATATCAGGCACTGAG
porcine GAPDH	GAPDH-F	TGATGACATCAAGAAGGTGGTGAAG
	GAPDH-R	TCCTTGGAGGCCATGTGGACCAT
PDCoV M	M-F	ATGTCTGACGCAGAAGAGTGGC

Target Gene	Primers	Primer Sequences（5’-3’）
	M-R	TTACATATACTTATACAGGCGAGCGTCA
PDCoV 3’UTR	3’UTR-F	CCATTTAATATGCCGAGGCCACG
	3’UTR-R	CCCCTAATTTGTCCCCACCACA
PDCoV N	N-F	ATGGCCGCACCAGTAGTC
	N-R	CTACGCTGCTGATTCCTGCTTTAT
PDCoV ORF1a	ORF1a-F	TTGGAGCCCAACCCACCTTG
	ORF1a-R	CCATTAACAGTGGTAGCCTTTGCC
PDCoV ORF1b	ORF1b-F	GGCACTCGAGGAGTCTATCAAGAAGT
	ORF1b-R	ATTGCTGCATGCGAAGACGC
