# Supplementary material for: Cellular eEF1G Inhibits Porcine Deltacoronavirus Replication by Binding Nsp12 and Disrupting Its Interaction with Viral Genomic RNA
Source: Viruses. 2025 Oct 13;17(10):1369. doi: 10.3390/v17101369 (PMC12568264; doi:10.3390/v17101369)
Supplement: Supplementary file 1 [file viruses-17-01369-s001.zip › Figure S1.pdf]

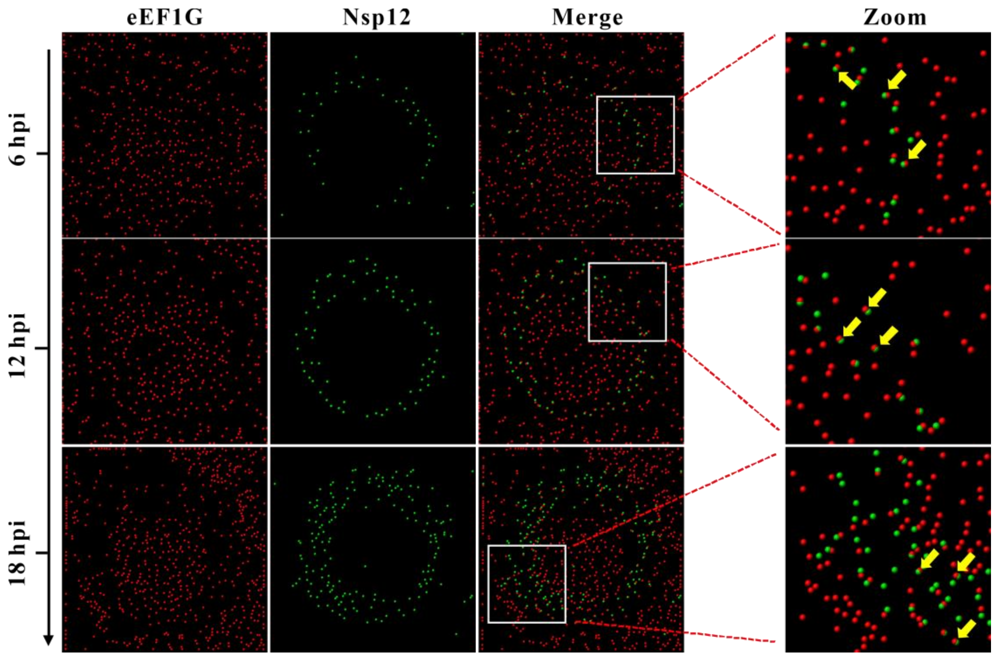

Figure S1. Three-dimensional reconstruction of eEF1G and Nsp12 co-localization in PDCoV-infected IPEC-J2 cells. The co-localization of eEF1G and Nsp12 in PDCoV-infected IPEC-J2 cells, presented in Figure 2B, was reconstructed using Imaris software. Pictures represent PDCoV Nsp12 protein (Green), eEF1G (Red), and merged images (Merge). The yellow arrows indicate sites of co-localization between eEF1G and Nsp12.
