# Supplementary material for: Cellular eEF1G Inhibits Porcine Deltacoronavirus Replication by Binding Nsp12 and Disrupting Its Interaction with Viral Genomic RNA
Source: Viruses. 2025 Oct 13;17(10):1369. doi: 10.3390/v17101369 (PMC12568264; doi:10.3390/v17101369)
Supplement: Supplementary file 1 [file viruses-17-01369-s001.zip › Figure S2.pdf]

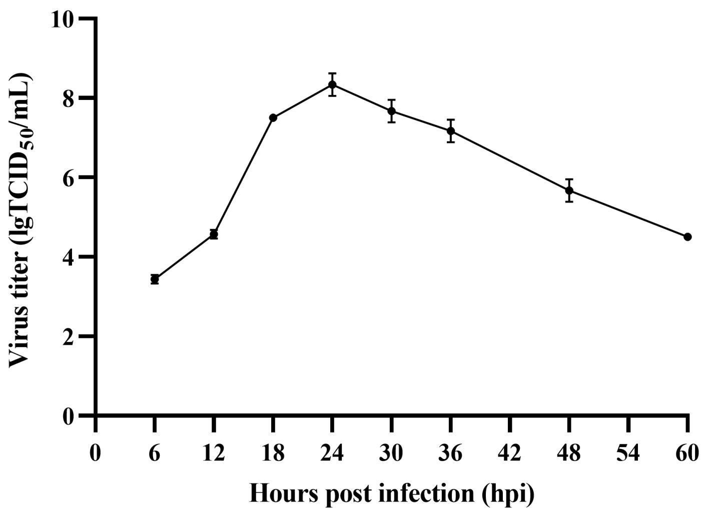

Figure S2. One-step growth curve of PDCoV in LLC-PK1 cells. LLC-PK1 cells were infected with PDCoV at an MOI of 5, then harvested at 6, 12, 18, 24, 30, 36, 42, 48, 54, and 60 hpi. Viral yields were determined by $TCID_{50}$ assay at different time points.
