# Supplementary material for: Cellular eEF1G Inhibits Porcine Deltacoronavirus Replication by Binding Nsp12 and Disrupting Its Interaction with Viral Genomic RNA
Source: Viruses. 2025 Oct 13;17(10):1369. doi: 10.3390/v17101369 (PMC12568264; doi:10.3390/v17101369)
Supplement: Supplementary file 1 [file viruses-17-01369-s001.zip › Figure S3.pdf]

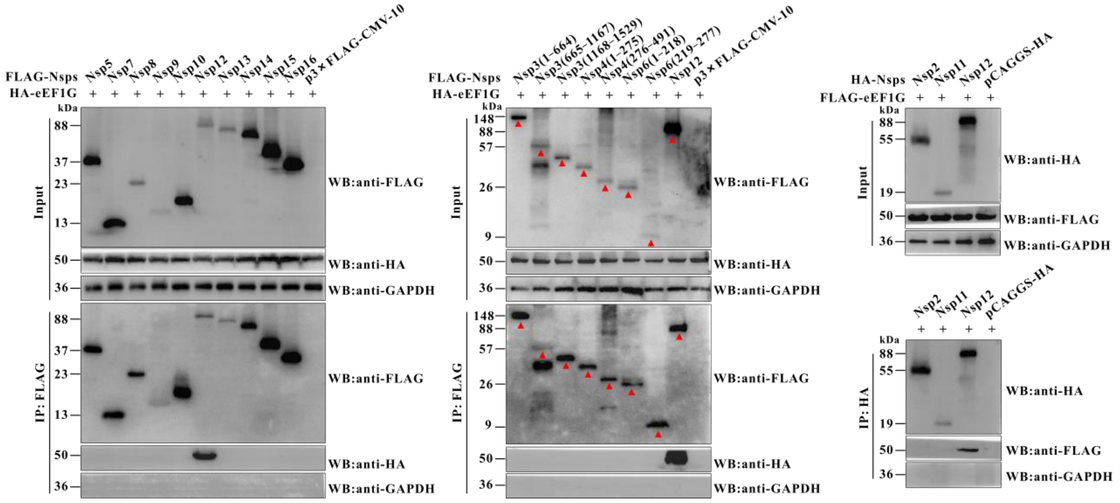

Figure S3. Co-IP analysis of the interactions between all PDCoV nonstructural proteins and cellular eEF1G protein. BHK-21 cells were co-transfected for 36 hours with the recombinant plasmid pCAGGS-HA-eEF1G (1 µg per well in a six-well plate) together with a p3×FLAG-CMV-10 plasmid (1 µg per well in a six-well plate) encoding an individual PDCoV Nsp or its truncation. Total cellular proteins were then extracted and subjected to Co-IP using anti-FLAG or rabbit anti-HA antibodies, using the empty pCAGGS-HA or p3×FLAG-CMV-10 plasmid as a control. Precipitated proteins were subsequently analyzed by western blotting, with target protein bands indicated by red triangles. “+” represents the presence of the indicated plasmid.
