# Supplementary material for: Cellular eEF1G Inhibits Porcine Deltacoronavirus Replication by Binding Nsp12 and Disrupting Its Interaction with Viral Genomic RNA
Source: Viruses. 2025 Oct 13;17(10):1369. doi: 10.3390/v17101369 (PMC12568264; doi:10.3390/v17101369)
Supplement: Supplementary file 1 [file viruses-17-01369-s001.zip › Figure S4.pdf]

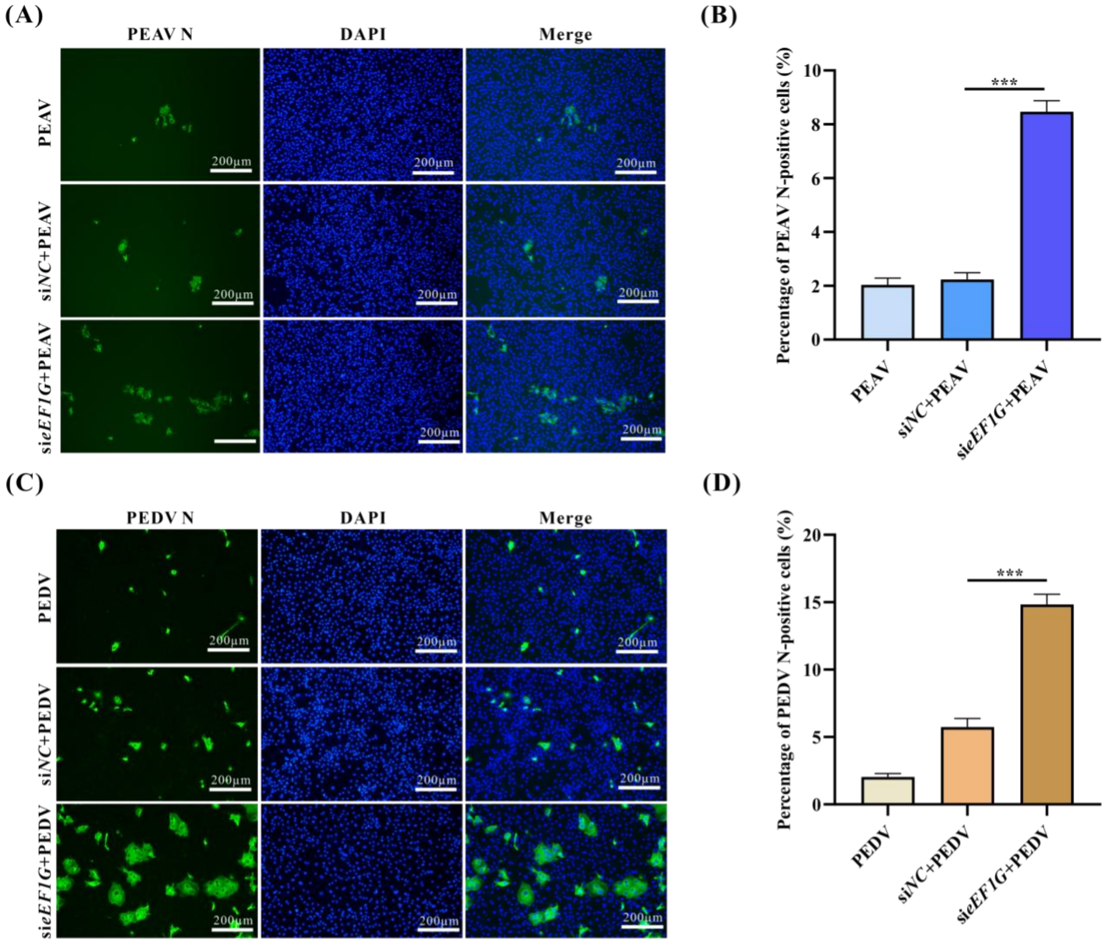

Figure S4. Knockdown of eEF1G significantly promotes PEAV and PEDV replication. (A) ST cells were transfected with either si*EEF1G* or scrambled control siRNA (si*NC*) for 36 h and then infected with PEAV at an MOI of 1. At 12 hpi, the cells were fixed and processed for confocal immunofluorescence analysis. After incubation with a primary antibody against PEAV N protein, the cells were treated with an Alexa Fluor 488-conjugated secondary antibodies. Nuclei were counterstained with DAPI. Pictures represent PEAV N protein (Green), nuclei (Blue), and merged images (Merge). Scale bar: 200 μm. **(B)** Statistical analysis of the percentage of N protein-positive cells among PEAV-infected ST cells shown in **(A)**. *** p < 0.001. **(C)** MARC-145 cells were transfected with either si*EEF1G* or scrambled control siRNA (si*NC*) for 36 h and then infected with PEDV at an MOI of 1. At 12 hpi, the cells were

fixed and processed for confocal immunofluorescence analysis. After incubation with a primary antibody against PEDV N protein, the cells were treated with an Alexa Fluor 488-conjugated secondary antibodies. Nuclei were counterstained with DAPI. Pictures represent PEDV N protein (Green), nuclei (Blue), and merged images (Merge). Scale bar: 200 μm. **(D)** Statistical analysis of the percentage of N protein-positive cells among PEDV-infected MARC-145 cells shown in **(C)**. *** $p < 0.001$.
