# Supplementary material for: Cellular eEF1G Inhibits Porcine Deltacoronavirus Replication by Binding Nsp12 and Disrupting Its Interaction with Viral Genomic RNA
Source: Viruses. 2025 Oct 13;17(10):1369. doi: 10.3390/v17101369 (PMC12568264; doi:10.3390/v17101369)
Supplement: Supplementary file 1 [file viruses-17-01369-s001.zip › Figure S5.pdf]

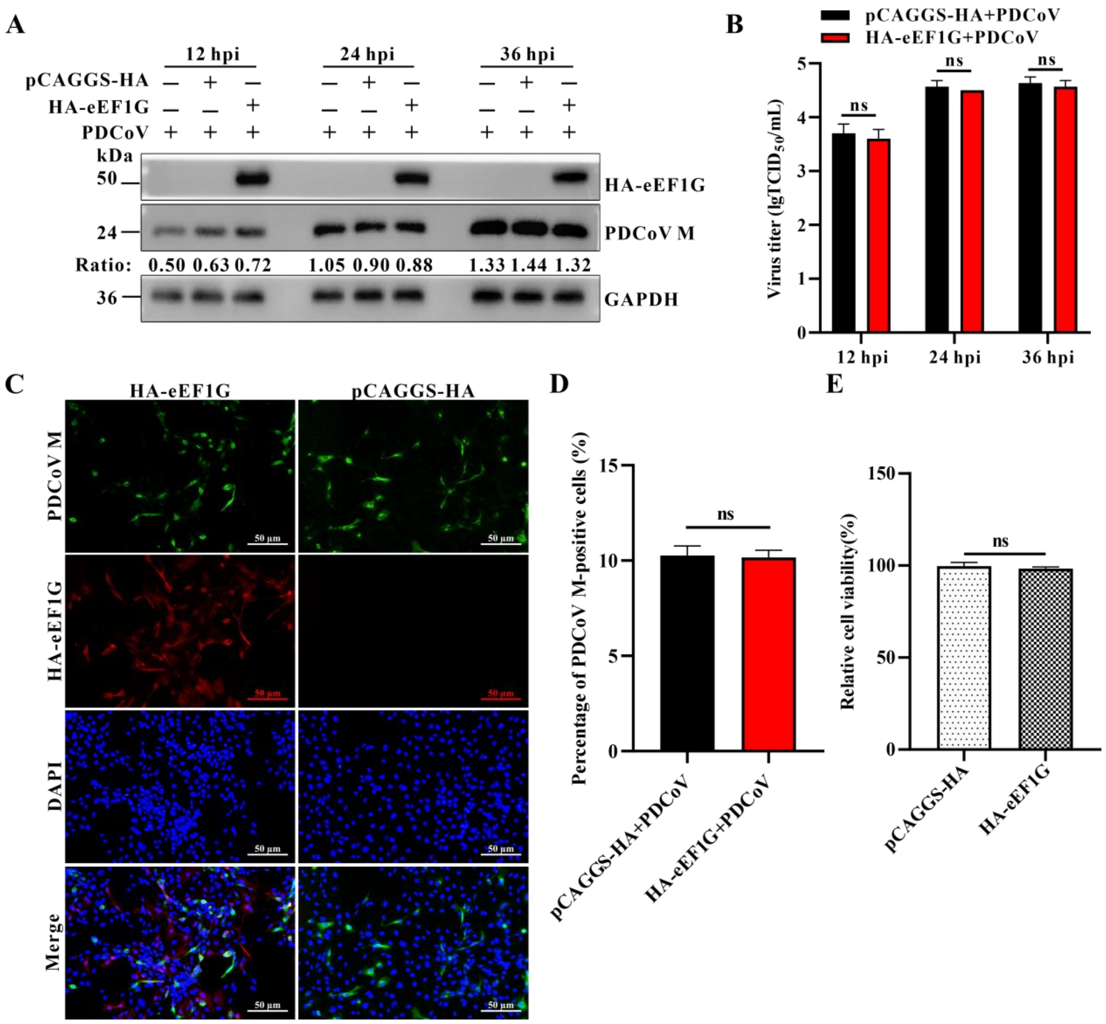

Figure S5. Overexpression of eEF1G does not significantly affect PDCoV replication in IPEC-J2 cells. (A) IPEC-J2 cells were transfected with either the recombinant plasmid pCAGGS-HA-eEF1G (2 μg per well in a six-well plate) or the empty vector control (2 μg per well in a six-well plate) for 36 h, followed by infection with PDCoV at an MOI of 1. Cells were harvested at 12, 24, and 36 hpi and subjected to western blot analysis using primary antibodies specific to PDCoV M protein, HA, and GAPDH (rabbit anti-HA antibody was used). The ratio of each target protein band intensity to that of the corresponding GAPDH loading control is indicated below the respective band. “+” represents the presence and “–” represents the absence of

indicated PDCoV or plasmids. **(B)** IPEC-J2 cells were transfected with either the recombinant plasmid pCAGGS-HA-eEF1G or the empty vector control for 36 h, followed by infection with PDCoV at an MOI of 1. Viral yields were determined by $TCID_{50}$ assay at 12, 24, and 36 hpi. Data, presented as means ± SD from three independent experiments, were analyzed by two-way ANOVA. ns, no significance. **(C)** IPEC-J2 cells were transfected with either the recombinant plasmid pCAGGS-HA-eEF1G or the empty vector control for 36 h, followed by infection with PDCoV at an MOI of 1. At 12 hpi, the cells were fixed and subjected to confocal immunofluorescence analysis. Primary antibodies specific to PDCoV M protein and the HA tag were applied (mouse anti-HA antibody was used), followed by incubation with Alexa Fluor 488-conjugated goat anti-rabbit IgG and Alexa Fluor 568-conjugated goat anti-mouse IgG secondary antibodies, respectively. Nuclei were counterstained with DAPI. Pictures represent PDCoV M protein (Green), HA-eEF1G (Red), nuclei (Blue), and merged images (Merge). Scale bar: 50 μm. **(D)** Statistical analysis of the percentage of M protein-positive cells among eEF1G-transfected and empty vector-transfected IPEC-J2 cells shown in **(C)**. ns, no significance. **(E)** Cell proliferation activity following eEF1G overexpression was assessed using the CCK-8 assay. Data are presented as mean ± SD from three independent experiments. ns, no significance.
