# Supplementary material for: Cellular eEF1G Inhibits Porcine Deltacoronavirus Replication by Binding Nsp12 and Disrupting Its Interaction with Viral Genomic RNA
Source: Viruses. 2025 Oct 13;17(10):1369. doi: 10.3390/v17101369 (PMC12568264; doi:10.3390/v17101369)
Supplement: Supplementary file 1 [file viruses-17-01369-s001.zip › Figure S6.pdf]

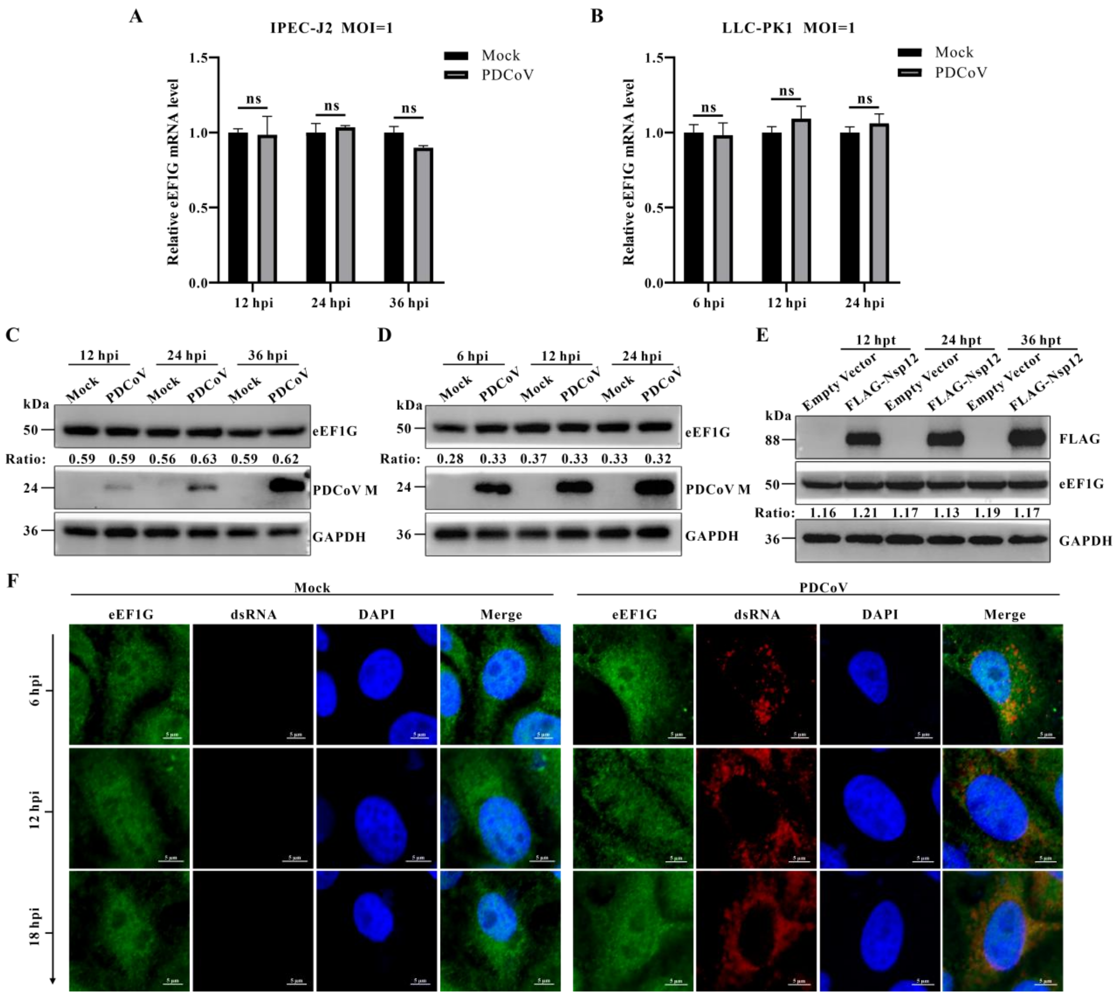

Figure S6. Cellular eEF1G abundance and localization are unchanged by PDCoV infection or Nsp12 expression. (A) IPEC-J2 cells were either mock-infected or infected with PDCoV (MOI=1), then harvested at 12, 24, and 36 hpi. The eEF1G mRNA levels were quantified by RT-qPCR using β-actin as a reference gene. Relative RNA levels at each time point were normalized to the mock-infected control. Data, presented as means ± SD from three independent experiments, were analyzed by two-way ANOVA. ns, no significance. **(B)** LLC-PK1 cells were infected and analyzed as described in **(A)**. **(C)** IPEC-J2 cells were either mock-infected or infected with PDCoV (MOI=1), then harvested at 12, 24, and 36 hpi. Whole-cell lysates were subjected to western blot analysis using antibodies against PDCoV M protein, eEF1G,

and GAPDH (rabbit anti-eEF1G antibody was used). The relative intensity of each target protein band, normalized to its corresponding GAPDH loading control, is indicated below each lane. **(D)** LLC-PK1 cells were infected and analyzed as described in **(C)**. **(E)** IPEC-J2 cells were transfected with either the recombinant plasmid p3×FLAG-CMV-10-Nsp12 (2 μg per well in a six-well plate) or its corresponding empty vector (2 μg per well in a six-well plate). At 12, 24, and 36 hour post-transfection (hpt), whole-cell lysates were prepared and analyzed by western blot using antibodies against FLAG, eEF1G, and GAPDH (rabbit anti-eEF1G antibody was used). The relative band intensity for each target protein, normalized to GAPDH, is indicated below each lane. **(F)** IPEC-J2 cells were mock-infected or infected with PDCoV (MOI=1). At 6, 12, and 18 hpi, cells were fixed and immunostained with primary antibodies against eEF1G and dsRNA (rabbit anti-eEF1G antibody was used), and then with corresponding Alexa Fluor 488-conjugated anti-rabbit and Alexa Fluor 568-conjugated anti-mouse secondary antibodies. Nuclei were counterstained with DAPI. Pictures represent eEF1G (Green), dsRNA (Red), nuclei (Blue), and merged images (Merge). Scale bar: 5 μm.
