# Supplementary material for: Cellular eEF1G Inhibits Porcine Deltacoronavirus Replication by Binding Nsp12 and Disrupting Its Interaction with Viral Genomic RNA
Source: Viruses. 2025 Oct 13;17(10):1369. doi: 10.3390/v17101369 (PMC12568264; doi:10.3390/v17101369)
Supplement: Supplementary file 1 [file viruses-17-01369-s001.zip › Figure S7.pdf]

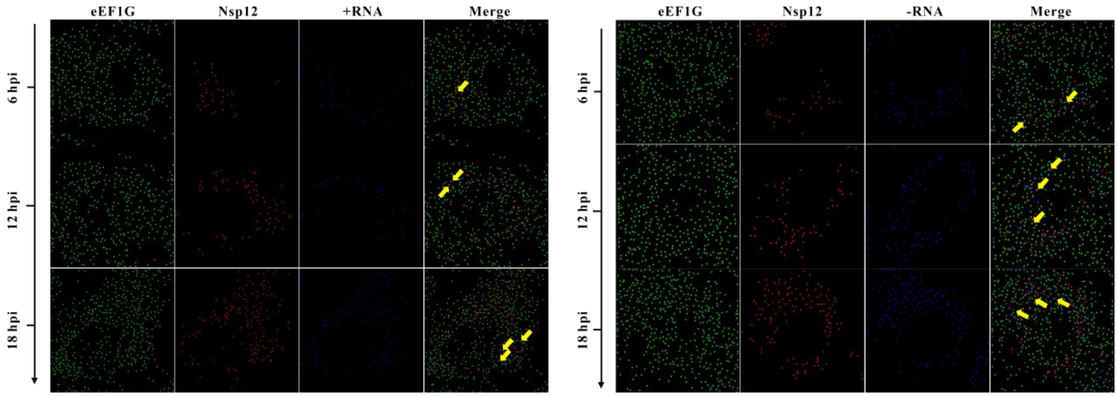

Figure S7. Three-dimensional reconstruction of the co-localization of eEF1G, Nsp12, and PDCoV RNA in infected IPEC-J2 cells. The co-localization of eEF1G, Nsp12, and PDCoV RNA in infected IPEC-J2 cells, presented in Figure 6A, was visualized using Imaris software, with yellow arrows marking the sites of colocalization. Pictures represent eEF1G (Green), PDCoV Nsp12 (Red), PDCoV RNA (Blue), and merged images (Merge).
