# Supplementary material for: Cellular eEF1G Inhibits Porcine Deltacoronavirus Replication by Binding Nsp12 and Disrupting Its Interaction with Viral Genomic RNA
Source: Viruses. 2025 Oct 13;17(10):1369. doi: 10.3390/v17101369 (PMC12568264; doi:10.3390/v17101369)
Supplement: Supplementary file 1 [file viruses-17-01369-s001.zip › Table S1.pdf]

Table S1. Primers used for plasmids construction

Primers	Primer Sequences（5'-3'）
HA-Nsp12-F	gttccagattacgctgaattcGAAATCCTGATCACAATGTGCG
HA-Nsp12-R	attaagatctgctagctcgagCTGCAGTGTGGGTGACTGTTCG
HA-eEF1G-F	gttccagattacgctgaattcATGGCGGCCGGGACCCTG
HA-eEF1G-R	attaagatctgctagctcgagTCACTTGAAGATCTTGCCCTGAT
HA-MX2-F	gttccagattacgctgaattcATGCCTAAACCCCGCATGT
HA-MX2-R	attaagatctgctagctcgagTTACATCCCTTGTACCTCAACCC
HA-LGALS1-F	gttccagattacgctgaattcATGGCTTGTGGTCTGGTCGC
HA-LGALS1-R	attaagatctgctagctcgagTCACTCAAAGGCCACACACTTG
HA-LGALS3-F	gttccagattacgctgaattcATGGCAGACGGTTTTTCGC
HA-LGALS3-R	attaagatctgctagctcgagTTATATCATAGTGTGTGAAGCACTGG TG
HA-HSPA6-F	gttccagattacgctgaattcATGTCCGCTGCAAGAGAAGTG
HA-HSPA6-R	attaagatctgctagctcgagTTAATCAACCTCCTCAATGACAGG
FLAG-Nsp12-F	gacgatgacaagcttgcggccgcGGAAATCCTGATCACAATGTGCG
FLAG-Nsp12-R	cctctagagtcgactggtaccCTGCAGTGTGGGTGACTGTTCG
GST-Nsp12-F	ttccaggggcccctgggatccGAAATTTTGATCACCATGTGCG
GST-Nsp12-R	gtcacgatgcggccgctcgagCTGTAGTGTAGGACTTTGCTCATAC ATC
Strep II -eEF1G-F	cagcaaatgggtcgcggatccTGGAGCCACCCGCAGTTCGAAAAA ATGGCGGCCGGGACCCTG

Primers	Primer Sequences（5’-3’）
Strep II-eEF1G-R	gcaagcttgtcgacggagctcTCACTTGAAGATCTTGCCCTGAT
eEF1G 408-F	gttccagattacgctgaattcATGGCGGCCGGGACCCTG
eEF1G 408-R	attaagatctgctagctcgagCTCCTCGCTGCCAGGATCCA
eEF1G 378-F	gttccagattacgctgaattcATGGCGGCCGGGACCCTG
eEF1G 378-R	attaagatctgctagctcgagTCGGAAGACCCAGACTCCAG
eEF1G 348-F	gttccagattacgctgaattcATGGCGGCCGGGACCCTG
eEF1G 348-R	attaagatctgctagctcgagTCGCTGGAACATTCCAGTGA
eEF1G 318-F	gttccagattacgctgaattcATGGCGGCCGGGACCCTG
eEF1G 318-R	attaagatctgctagctcgagGCCATCCTTATCAAAGTGCT
eEF1G 271-F	gttccagattacgctgaattcATGGCGGCCGGGACCCTG
eEF1G 271-R	attaagatctgctagctcgag AGCCAGCGCCTGCTCA
eEF1G 437-F	gttccagattacgctgaattcGCTGCTGAGCCCAAGGCC
eEF1G 437-R	attaagatctgctagctcgagCTGATTGAAGGCTTTGCCCA
Nsp12 665-F	gacgatgacaagcttgcggccgcGGAAATCCTGATCACAATGTGCG
Nsp12 665-R	cctctagagtcgactggtaccCTGTGAGCAGAACTCGTGGGG
Nsp12 529-F	gacgatgacaagcttgcggccgcGGAAATCCTGATCACAATGTGCG
Nsp12 529-R	cctctagagtcgactggtaccTCCTCCGGGCTTCACGTAC
Nsp12 432-F	gacgatgacaagcttgcggccgcGGAAATCCTGATCACAATGTGCG
Nsp12 432-R	cctctagagtcgactggtaccCAGTGAGATTGACTTCAGCATCTTC
Nsp12 781-F	gacgatgacaagcttgcggccgcGATCAACCTGAACACACTGCTGA
Nsp12 781-R	cctctagagtcgactggtaccCTGCAGTGTGGGTGACTGTTCG

Primers	Primer Sequences（5’-3’）
Nsp12 218-F	gacgatgacaagcttgcggccgcGGAAATCCTGATCACAATGTGCG
Nsp12 218-R	cctctagagtcgactggtaccGTTTGCCATGTGTGTTGTCACG
FLAG-Nsp5-F	gacgatgacaagcttgcggccgcGGCTGGAATTAAAATCCTGCTG
FLAG-Nsp5-R	cctctagagtcgactggtaccCTGGAGGCTAATAGGGGCCT
FLAG-Nsp7-F	gacgatgacaagcttgcggccgcGAACAAGATCCTGGACGCCA
FLAG-Nsp7-R	cctctagagtcgactggtaccCTGGACGACGGCCTTGTTT
FLAG-Nsp8-F	gacgatgacaagcttgcggccgcGGCTGTTGCCGATATCAACAT
FLAG-Nsp8-R	cctctagagtcgactggtaccTTGCAACTGGGGCACACC
FLAG-Nsp9-F	gacgatgacaagcttgcggccgcGAACAATGAGCTATGTTTGCGC
FLAG-Nsp9-R	cctctagagtcgactggtaccCTGAAGGATAGTAGTTTCAGAGATG TGG
FLAG-Nsp10-F	gacgatgacaagcttgcggccgcGGCAAGTGGCACTCAAATTGA
FLAG-Nsp10-R	cctctagagtcgactggtaccCTGCAGACTAGATCCACAGGTGC
FLAG-Nsp13-F	gacgatgacaagcttgcggccgcGGCCTCCGGAGTGTGCGT
FLAG-Nsp13-R	cctctagagtcgactggtaccCTGCAGCTGGGAGTCGATGG
FLAG-Nsp14-F	gacgatgacaagcttgcggccgcGAGCAGCGCAGAGAAAAACC
FLAG-Nsp14-R	cctctagagtcgactggtaccCTGGAGGTTGGTGAAGGTCAGC
FLAG-Nsp15-F	gacgatgacaagcttgcggccgcGAACCTGGAAAACCTGGCCT
FLAG-Nsp15-R	cctctagagtcgactggtaccCTGGAGGATGGGATAGCAGGT
FLAG-Nsp16-F	gacgatgacaagcttgcggccgcGAGCCTGACCAACGGGTATCA
FLAG-Nsp16-R	cctctagagtcgactggtaccTCAATAGAGCTCTCGGCATTCA

Primers	Primer Sequences（5'-3'）
FLAG-Nsp3(664)-F	cctctagagtcgactggtaccGGTCACTTCGAGGTAAATCTGGC
FLAG-Nsp3(664)-R	cctctagagtcgactggtaccGGTCACTTCGAGGTAAATCTGGC
FLAG-Nsp3(1167)-F	gacgatgacaagcttgcggccgcGGAGGAAGTCCAGAACCAGGC
FLAG-Nsp3(1167)-R	cctctagagtcgactggtaccATAGAAATAGTACACGCTGAAGGTCATC
FLAG-Nsp3(1529)-F	gacgatgacaagcttgcggccgcGAAAGTCATGAAATTCTTCCGCC
FLAG-Nsp3(1529)-R	cctctagagtcgactggtaccGGCGAAGTACACAATGGCGA
FLAG-Nsp4(275)-F	gacgatgacaagcttgcggccgcGGCTATGGCCTTCGGCTT
FLAG-Nsp4(275)-R	cctctagagtcgactggtaccGTGGATGCCGGACACGGT
FLAG-Nsp4(491)-F	gacgatgacaagcttgcggccgcGATCTTCACCAGCACCGCC
FLAG-Nsp4(491)-R	cctctagagtcgactggtaccCTGCAGTTTGGTCTTCAGGGA

Primers	Primer Sequences（5’-3’）
FLAG-Nsp6(218)-F	gacgatgacaagcttgcggccgcGTCAGGAGTAGTTAAGAAAACTTGTACGT
FLAG-Nsp6(218)-R	cctctagagtcgactggtaccTGCAAGCCACATAAGGCCA
FLAG-Nsp6(277)-F	gacgatgacaagcttgcggccgcGAATCGATTCACAACTGTACCTATGG
FLAG-Nsp6(277)-R	cctctagagtcgactggtaccTTGGACAGTGGAAACAGCAATG
HA-Nsp2-F	gttccagattacgctgaattcATGGCCAAGAACAAGTCCAAGC
HA-Nsp2-R	attaagatctgctagctcgagACCAGCAACTTTCCGAAGGAA
HA-Nsp11-F	gttccagattacgctgaattcAATTCGGCTTATTTAAACCGAGTAA
HA-Nsp11-R	attaagatctgctagctcgagTTGAATAACGTCCTTAGATGTTGACA
FLAG- eEF1G-F	gacgatgacaagcttgcggccgcATGGCGGCCGGGACCCTG
FLAG- eEF1G-R	cctctagagtcgactggtaccTCACTTGAAGATCTTGCCCTGAT
